# Case Report: Bronchogenic cysts with pericardial hypoplasia are directly supplied by the descending aorta

**DOI:** 10.3389/fmed.2026.1709492

**Published:** 2026-02-05

**Authors:** Mingyang Zang, Yue Zhao, Chaoliang Yang, Weiwei Lv, Hao Wang, Xiangchen Liu, Xiankun Xue, Zhenguo Sun

**Affiliations:** 1 Department of Thoracic Surgery, Qilu Hospital of Shandong University, Jinan, Shandong, China; 2 Department of Thoracic Surgery, Xishui County People's Hospital, Zunyi, Guizhou, China; 3 Department of Radiology, Qilu Hospital of Shandong University, Jinan, Shandong, China; 4 Department of Pathology, Qilu Hospital of Shandong University, Jinan, Shandong, China

**Keywords:** bronchogenic cyst, aorta blood supply, pericardium hypoplasia, linear cutting stapler, preoperative vascular assessment

## Abstract

Bronchogenic cysts (BCs) are a type of congenital mediastinal tumor. This article reports a case that a BC associated with pericardial hypoplasia receives direct arterial supply from the descending aorta, forming a structure resembling pulmonary sequestration. A 17-year-old female was accidentally discovered to have a mediastinal cyst during a routine physical examination. The patient was asymptomatic. Computed tomography (CT) imaging revealed that there was a thick blood vessel on the surface of the cyst, which directly connected to the descending aorta. Following comprehensive preoperative vascular assessment, the cyst was successfully resected intraoperatively by linear cutting stapler, minimizing the risk of significant perioperative hemorrhage. Additionally, partial hypoplasia of the left pericardium was found during the operation, which was suspicious to be related to the BC. Given the limited residual pleural space following cyst removal, pericardial suturing was not performed. This article aims to provide ideas and references for the subtypes and treatment methods of BCs in the future, and emphasizes the importance of preoperative vascular assessment.

## 1 Introduction

BCs are congenital lesions that result from abnormal budding of the ventral foregut during embryogenesis (1). They are most frequently located in the middle or posterior mediastinum near the tracheal bifurcation. BCs are typically asymptomatic, but they still can cause some complications. Surgical resection is the recommended treatment method (2). BCs associated with pericardial hypoplasia are extremely rare. Some literature has reported such cases (1, 3), but to date, there has been no case where BCs are directly supplied by the descending aorta and resected using a linear cutting stapler. This case report presents a patient with a BC with pericardial hypoplasia which is directly supplied by the descending aorta, and aims to extend the understanding of BCs and offer a referable treatment option.

## 2 Case data

**Figure 1:**
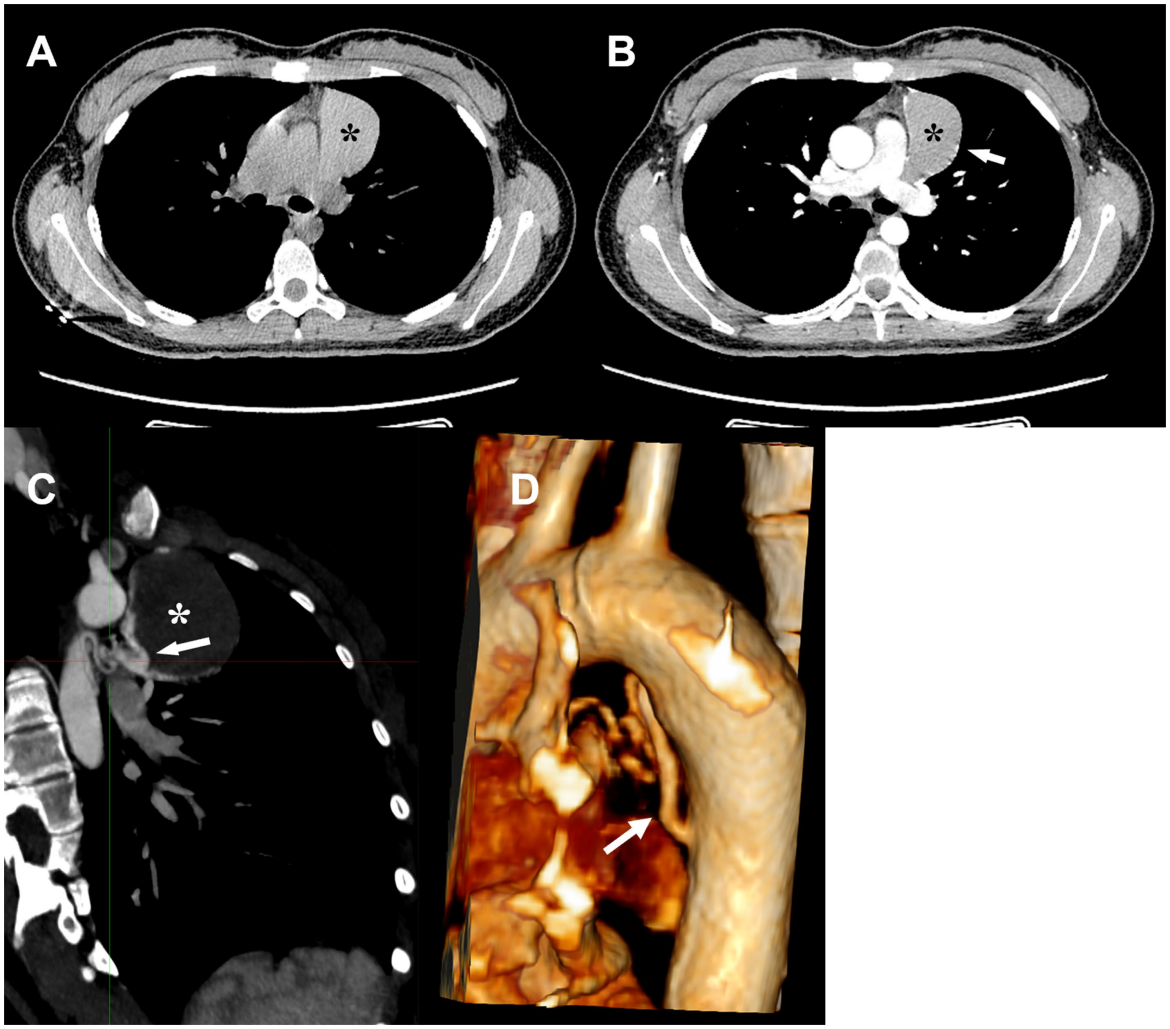
Compared with the CT scan without contrast **(A)**, the cyst wall was enhanced on contrast-enhanced images **(B)**. Sagittal CT image **(C)** and 3D reconstruction image **(D)** demonstrated a feeding vessel originating from the descending aorta and supplying the cyst. Black and white *: cyst, white arrowhead: branch artery of the descending aorta.

A 17-year-old female was incidentally found to have a mediastinal mass on the chest CT during a routine physical examination 5 days prior to consultation. No respiratory or systemic symptoms and no past medical history. A chest CT scan showed a nearly circular soft-tissue density and fluid-filled lesion in the anterior superior mediastinum on the soft tissue density window (Figure 1A). On chest contrast-enhanced CT, there was no visible enhancement of the contents inside the cyst, but the edge of the cyst wall was significantly enhanced (Figure 1B). A sagittal CT scan and three-dimensional reconstructive CT scan also demonstrated a curved vascular structure extending from the aorta to the cyst (Figures 1C,D). The CT scans were obtained with a 64-slice spiral CT machine, 5.5-mm slice thickness and pitch 1.5, before and after i.v. administration of contrast agent. Three-dimensional reconstructed CT images were generated utilizing both Multi-Planar Reconstruction (MPR) and Volume Rendering Technology (VRT). Complete blood count was within normal limits and no notable abnormal basic metabolic panel parameters emerged. No abnormalities were found during physical examination.

## 3 Treatment

**Figure 2:**
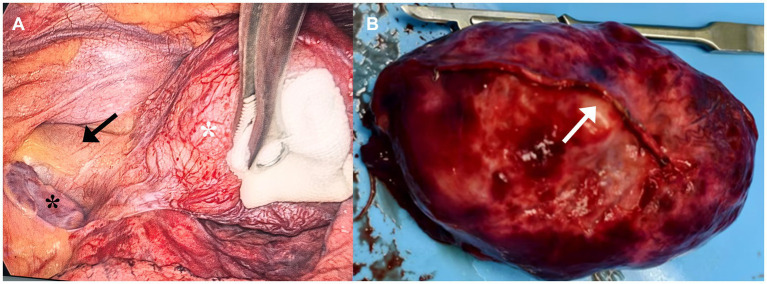
Medical thoracoscopic view **(A)**. Black *: The left atrium exposed outside the pericardium, white *: cyst, black arrowhead: aorta. Resected cyst with visible blood vessels **(B)**. White arrowhead: visible artery locating on the surface of the cyst.

**Figure 3:**
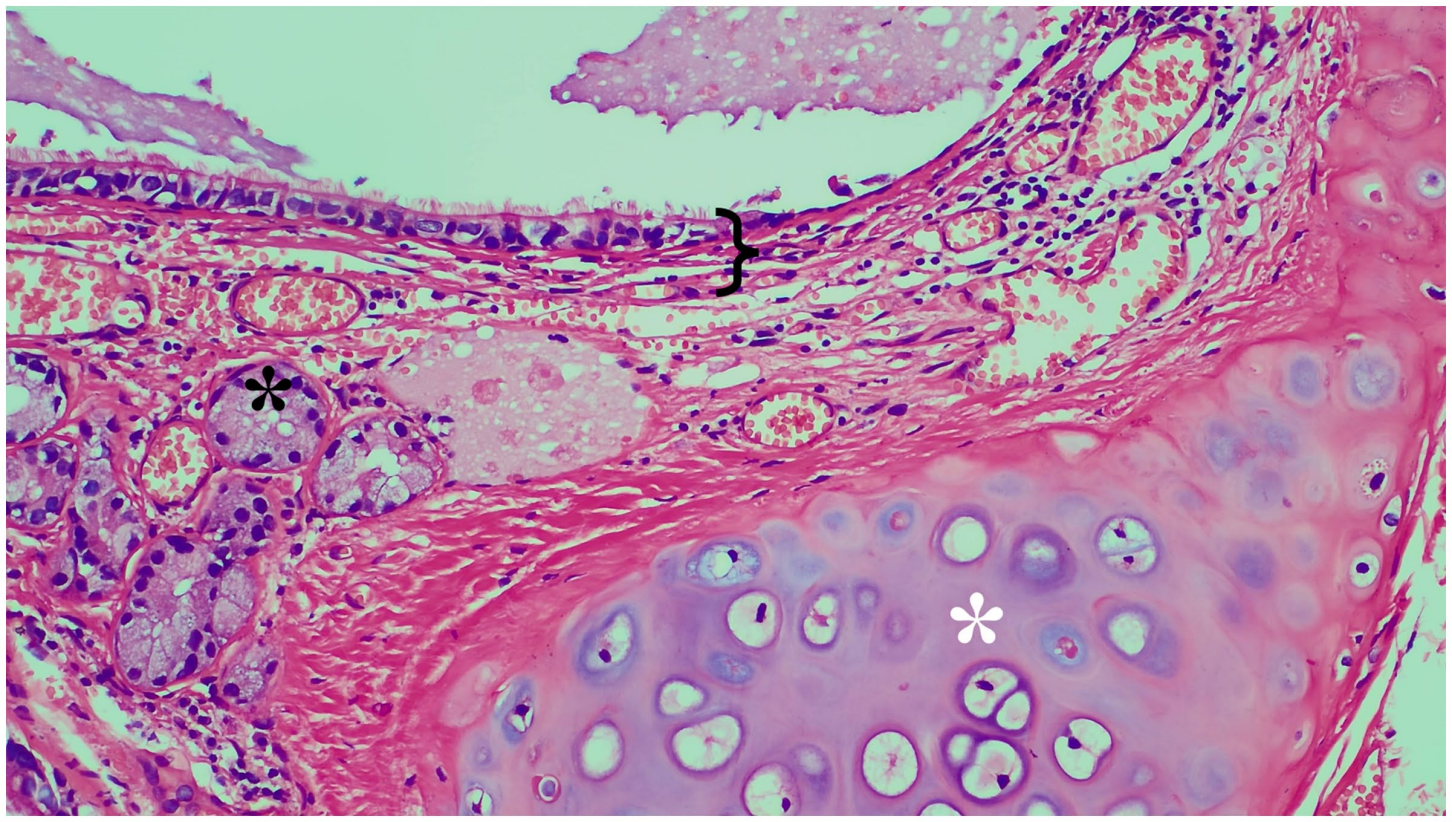
Histopathological photomicrograph of the cyst (HE staining, 20 × magnification). Black callout brace: bronchial epithelium and the lamina propria. Black *: bronchial gland. White *: hyaline cartilage.

**Figure 4:**
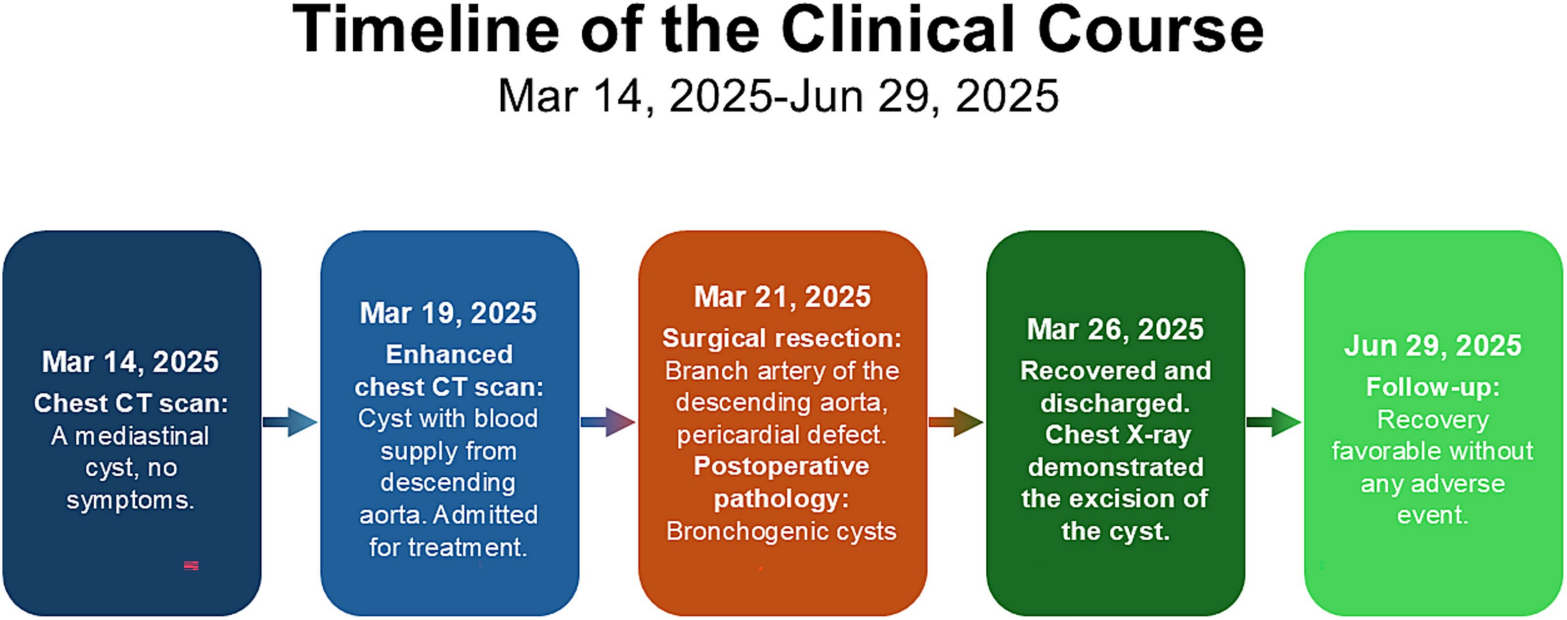
Timeline of the clinical course.

Based on these examination and test results, the patient was initially diagnosed with a BC, and it is highly suspicious that the aorta directly supplies blood to it. Although the patient showed no symptoms, she still underwent surgical treatment. To minimize the risk of intraoperative hemorrhage, a linear cutting stapler was selected for the cyst resection instead of the traditional ultrasonic scalpel or electrocautery. In fact, even after resecting the cyst with the linear cutting stapler, persistent bleeding was observed at the resection margin. Hemostasis was ultimately achieved only after sustained compression with gauze for approximately 5 min. Meanwhile, partial hypoplasia of the left pericardium was also found during the operation, but no suture treatment was performed (Figure 2A). Pathological examination of the cyst removed by the operation demonstrated a benign BC (Figure 3), with a clear arterial vessel noted on its surface after resection (Figure 2B). The operation lasted for 1h35min which the patient’s condition kept stable. The blood loss during the operation was about 50 mL. Electrocautery was used for hemostasis of superficial venous bleeding and suture ligation was for deep venous or arterial bleeding. No blood transfusion was performed during or after the operation. The patient did not experience any adverse reactions after the operation. On the first day after the operation, a chest X-ray scan was performed for re-examination, indicating that cyst had been removed. On the third day after the operation, the thoracic drainage tube was removed. During the drainage period, the color of the drainage fluid was normal, and the volume gradually decreased day by day. The patient was discharged on the fourth day after the operation. The patient’s postoperative condition remained stable without recurrence during the follow-up after 3 months after operation, and physical examinations of the lungs and other parts showed no abnormalities (Figure 4).

## 4 Discussion

BCs on CT images may exhibit fluid-like density, soft tissue-like density, or solid density depending on their location and contents, but none of them own large vessels. It’s also challenging to distinguish the cysts from other diseases such as thymomas, thymic cysts et al. when they manifest as soft tissue-like density on CT images and pathological examination is required (4). Although BCs are benign lesions, they carry the risk of complications such as infection, hemorrhage, or, in rare instances, malignant transformation. Therefore, surgical resection is typically recommended for treatment (2). In this report, CT imaging revealed an enhancement on cyst wall, a feature that has not been commonly described in previous reports. This raised the suspicion of an associated vascular supply to the lesion. We chose the linear cutting stapler as the surgical method which is suitable for surgical scenarios, where rapid and tight closure of the lumen is required, such as in large blood vessel dissection, lung lobe resection, subtotal gastrectomy, intestinal anastomosis etc. (5). Surgical process proves the correction of our decision.

Pulmonary sequestration consists of a mass of nonfunctional lung tissue supplied by one or more systemic arteries, whose structure closely resembles the cyst in this case, necessitating careful differentiation during diagnosis. Chest CT manifestation of pulmonary sequestration can vary among cystic, cystic-gas, cystic-solid and solid masses. Meanwhile, thick abnormal blood supply arteries can be displayed on CT enhancement images which is different from BCs (6). The common pathological features of the pulmonary sequestration often show dysplasia of lung tissue, with irregular cyst and secretion, chronic inflammatory reaction with fibrosis in septa, and abnormal or occluded blood vessels (7). Conversely, BCs are composed of cup-shaped cells filled with mucin and ciliated pseudostratified columnar epithelium, that smooth muscle and even cartilage can appear on the wall of the cyst (8).

It is reported that various congenital cardiac abnormalities are associated with BCs, such as incomplete pericardial development, patent ductus arteriosus, mitral valve stenosis, etc. (3). Congenital pericardial hypoplasia is an extremely rare disease, with an incidence of less than 1 case per 10,000 people in the population, and most patients have no symptoms (9). Many articles have reported cases of BCs accompanied by pericardial hypoplasia, but none of them focus on the possible common causes that might exist between them. BC is typically caused by abnormal budding of the tracheobronchial tree that occurs during the 10–12 weeks of gestation, while congenital pericardium hypoplasia results from abnormal fusion of the pleura and pericardium which occur at the same time below (10). Given the adjacent anatomical locations of the tracheobronchial tree and the pleuropericardial membrane during the embryonic stage, and the consistency in their developmental time, we hypothesize that these two diseases originated from an early common development disorder in this location and mutually influenced each other. At the same time, in the absence of pericardial isolation, the abnormal BCs may contact with the early arterial trunk directly and induce the formation of a branch artery to supply blood to it.

In our case, the remaining space after cyst excision was minimal and typically filled by the expanding lung parenchyma (1). The patient had not previously shown any symptoms suggestive of pericardial insufficiency. Therefore, we chose not to perform pericardial suturing. The patient’s postoperative course supported our decision.

This case presents a new vascular variation type of BCs that have not been reported before, providing new evidence for the discovery of subtypes and new perspectives for the diagnosis and treatment of BCs. Meanwhile, this case highlights the critical importance of preoperative vascular assessment in mediastinal cysts with atypical enhancement features and supports the use of linear cutting stapler as a safe and effective tool in managing highly vascularized lesions.

Unfortunately, this report did not delve deeply into the potential mechanism by which BCs affect the development of the pericardium and aorta. In the future, we will continue to pay attention to this type of vascular variation to prove the universality of this subtype and explore the embryological mechanism by which BCs affect the development of the pericardium and aorta.

## 5 Patient perspective

At the 3-month follow-up visit after the surgery, the patient reported that she was extremely happy with the success of the operation. She did not feel any discomfort currently. She sincerely hoped that this report can help more people like her.

## Data Availability

The original contributions presented in the study are included in the article/supplementary material, further inquiries can be directed to the corresponding author.
